# Fear of falling in obese women under 50 years of age: a cross-sectional study with exploration of the relationship with physical activity

**DOI:** 10.1186/s40608-019-0230-z

**Published:** 2019-03-04

**Authors:** Gilly Rosic, Anne M. Milston, Jim Richards, Paola Dey

**Affiliations:** 1 Nepean Blue Mountains Family Metabolic Health Service, Nepean Clinical School, Kingswood, New South Wales and University of Sydney, Sydney, Australia; 2 Faculty of Health and Wellbeing, University of Central Lancashire, Preston, Lancashire UK; 3 Allied Health Research Unit, University of Central Lancashire, Preston, Lancashire UK; 4 Faculty of Health and Social Care, Edge Hill University, Road, Ormskirk, St Helens, Lancashire L39 4QP UK

**Keywords:** Obesity, Fear of falling, Physical activity, Postural balance, Self-efficacy

## Abstract

**Background:**

An understanding of capacity for physical activity in obese populations should help guide interventions to promote physical activity. Fear of falling is a phenomenon reported in the elderly, which is associated with reduced mobility and lower physical activity levels. However, although falls are reportedly common in obese adults, fear of falling and its relationship with activity has not been investigated in younger obese populations.

**Methods:**

In a cross-sectional study, fear of falling was measured in 63 women aged 18 to 49 years, with mean BMI 42.1 kg/m^2^ (SD 10.3) using the Modified Falls Efficacy (MFES), the Consequences of Falling (COF) and the Modified Survey of Activities and Fear of Falling in the Elderly (MSAFFE) scales. The choice of scales was informed by prior qualitative interviews with obese younger women. Physical activity levels were measured at the same time using the International Physical Activity Questionnaire. The mean score for fear of falling scales, with 95% confidence intervals, were estimated. Chi-square tests and t-tests were used to explore differences in age, body mass index and fear of falling scores between fallers and non-fallers. For each fear of falling scale, binomial logistic regression was used to explore its relationship with physical activity.

**Results:**

Mean scores suggested high levels of fear of falling: MFES [mean 7.7 (SD 2.7); median 8.5]; COF [mean 31.3 (SD 9.4)]; MSAFFE [mean 25.9 (SD 8.7); median 23]. Scores were significantly worse in fallers (*n* = 42) compared to non-fallers (*n* = 21). MFES and MSAFFE were independently associated with lower levels of physical activity [odds ratio = 0.65, 95% Cl 0.44 to 0.96 and odds ratio = 1.14, 95% CI 1.01 to 1.28 respectively], when adjusted for age, BMI and depression.

**Conclusions:**

This study confirms that fear of falling is present in obese women under 50 years of age. It suggests that it is associated with low levels of physical activity. These novel findings warrant further research to understand capacity for physical and incidental activity in obese adults in both genders and suggest innovative interventions to promote lifestyle changes and/or consideration of falls prevention in this population.

## Background

Increasing physical activity in obese adults is a key component of weight management for adults who are obese [1]. However, compliance with physical activity interventions is low. Lack of motivation is not the only barrier to physical activity in obese adults, who also report other concerns such as musculoskeletal pain, fear of movement and low mood [2, 3]. In addition, obese adults are more likely to report falls and balance problems [4–6]. These concerns are also seen in the elderly with ‘fear of falling’, a phenomenon that has been inconsistently defined but encompasses a number of related concepts, such as, lack of confidence in undertaking activities because of concerns about falling, activity avoidance because of concerns about falling and concerns about the consequences of falling [7]. In the elderly, ‘fear of falling’ has been shown to lead to mobility restrictions and reduced physical activity, both among those who have fallen and among those who have not, particularly in those with a body mass index over 40 [8–10]. There is an increasing body of literature showing increased levels of ‘fear of falling’ in other conditions that can affect balance, such as, stroke, or are associated with chronic musculoskeletal pain, such as, fibromyalgia [11, 12]. The primary objective of this study was to measure fear of falling levels, using validated tools, in younger obese adults (under the age of 50 years). We expected that younger obese adults would have elevated fear of falling as measured by the validated tools. We also explored whether greater fear of falling was associated with lower levels of physical activity in this population.

## Methods

This was a cross-sectional study. We recruited from weight management services in North West England. We included women aged 18 to 49 years of age with a body mass index over 30 kg/m^2^. We excluded women with known conditions that could affect their balance from the study. Weight management staff approached women to take part and, if they agreed, the participant self-completed an anonymised questionnaire, returned by post. Weight management staff recorded women’s most recent body mass index, using weight and height as measured in the clinic, on this questionnaire. The questionnaire included questions on the frequency of falls in the last 12 months; activity levels measured using the short form International Physical Activity Questionnaire (SF-IPAQ) [13]; mood measured using the subscales of the Hospital Anxiety and Depression Scale (HADS) [14], and three questionnaires covering different aspects of fear of falling. The SF-IPAQ is well validated and has been used to measure activity levels in populations who are obese [15, 16]; established thresholds were used to categorise participants into one of three activity level groups using well-established cut-offs: low, moderate or high [13]. Verbal consent was obtained from the participant as they self-completed the questionnaire and return of the anonymised questionnaire was evidence of implied consent; this was approved by the ethics committee. Ethics committee approval stipulated collection of age group, rather than age year, to facilitate anonymity. We had no permission to identify potentially eligible participants.

Choice of fear of falling scales: There are no fear of falling scales developed for adults who are obese. Therefore, the choice of fear of falling scales was guided by interviews undertaken previously by the lead author with 12 women under 50 years of age with BMI above 30 kg/m^2^ from the same weight management services [17], and by a systematic review of fear of falling scales developed for elderly populations [18]. No single scale covered all the concerns raised by the interviewees, so three questionnaires were chosen to cover different aspects.

*Modified Falls Efficacy Scale*: In the interviews, women frequently mentioned that they had concerns about falling when getting in and out of bed, using public transport, and using steps outside the house. These activities are in the Modified Falls Efficacy Scale (MFES) [19]. There are 14 questions on different activities in the MFES. Each item is answered on a scale of one to 10, in which one is not confident at all and 10 is completely confident. An overall score is estimated by adding the scores for each item and dividing by the number of items; a lower mean score suggests more fear of falling; a threshold of less than 8 has been used to denote presence of fear of falling [19].

*Consequence of Falling Scale:* Women in the interviews raised concerns about the consequences of falling; these included concerns about injury and pain but also about feeling foolish or embarrassed. Therefore, the questionnaire included the Consequence of Falling Scale (COF) [20]. This scale measures how worried people are about 12 different consequences if they fall over, and includes questions on worries about pain, severe injury, feeling foolish and being embarrassed. Each question is answered on a Likert-type scale (strongly disagree /disagree /agree /strongly agree), and scored one to 4 with the higher score denoting more problem. The scores are added up across the scale to give a total score and a score for each of two subscales (6 questions each): Loss of Functional Independence and Damage to Identity.

*Modified Survey of Activities and Fear of Falling in the Elderly Scale:* Some interview participants reported on how their concerns about falling, or its consequences, had led to a reduction in undertaking activity or avoidance of exercise or leisure-time activities. Therefore, the Modified Survey of Activities and Fear of Falling in the Elderly Scale (MSAFFE) was included as this covers whether people would never, sometimes or always avoid 17 different activities [20, 21]. The item scores (1 = ‘never avoid’, 2 = ‘sometimes avoid’, 3 = ‘always avoid’) are added to produce a total score.

For both the latter scales, a higher score denotes more fear of falling but there are no reported thresholds. All the chosen scales have high internal consistency, but only the MFES has excellent test-retest reliability [18].

Sample size: there was limited information to inform sample sizes for this study. There were two studies reporting the mean MFES in elderly fallers [19, 22]. The standard deviations (SD) of the mean MFES in these studies were 1.68 and 2.21. To achieve a precision of +/− 0.5 around the mean with 95% confidence, a sample size of either 75 or 43 respectively was needed. The study aimed to achieve at least the lower estimate and no more than the higher estimate. Participant recruitment commenced in 2014 for six months.

Analysis: in the event of one or two missing values on the HADS or a fear of falling scale, the total score was imputed based on the other values. The mean (SD) of fear of falling scales, and subscales, with 95% confidence intervals (CI) were estimated. Chi-square tests and t-tests were used to explore differences in age, body mass index and fear of falling scores between fallers and non-fallers. For each fear of falling scale, binomial logistic regression was used to explore its relationship with physical activity. Analysis was limited to women with a full dataset for the variables of interest. Separate analyses were undertaken for each scale because the approach was exploratory and there is potentially overlap between the different scales. This was supported by high correlations between the scales (r = − 0.56 for MFES and COF, r = − 0.76 for MSAFFE and MFES and r = 0.81 for COF and MSAFFE). Age and body mass index were included in the analyses, as these variables are associated with lower levels of physical activity [23]. Physical activity was collapsed into two subgroups (low activity and moderate/high activity) as the concern in this population is about low levels of activity. Age groups were collapsed into three groups (18 to 34 years, 35 to 44 years and 45 years and over) as this these groupings have some context in terms of life course and functional decline, e.g., early adulthood (18 to 34 years) and peri menopause/menopause (45 years and over) [24]. This strategy also ensured sufficient numbers in each category for analytical purposes. Body mass index was included as a continuous variable. Anxiety and depression subscales of the HADS were highly correlated (r = 0.77, *P* < 0.001) and anxiety is considered a key component of fear of falling [25]. Therefore, only the depression subscale scores, as a continuous variable, were included in the regression analyses. The mediating impact of a previous history of self-reported falls was also explored.

## Results

In total, 63 women agreed to participate in the study. Their characteristics are shown in Table 1. Twenty-six (41%) women were under 40 years of age and body mass ranged from 30.0 to 76.6 kg/m^2^ (mean 42.1 kg/m^2^ (SD10.3) (Table 1). Anxiety levels were high (mean 10.1 (SD 4.8)). In total, two-thirds (*n* = 42, 67%, 95% CI 56% to 79%) reported that they had fallen in the previous 12 months, of whom 28 (67%) had fallen more than once. Of the 63 participants, 58 (92%) completed the SF-IPAQ adequately to assign activity level, of whom 26 (45%) had low activity levels. The mean scores on the three fear of falling scales are also in Table 1.

**Table 1:** Characteristics of 63 women participating in the study

Characteristic	
Age in years n (%)	
18 to 24	3 (4.8%)
25 to 29	6 (9.5%)
30 to 34	9 (14.3%)
35 to 39	8 (12.7%)
40 to 44	13 (20.6%)
45 to 49	24 (38.1%)
Body mass index kg/m^2^ n (%)	
30–34.9	16 (24.4%)
35–39.9	17 (27.0%)
40–44.9	17 (27.0%)
> 45	13 (20.6%)
Mean (SD)	42.1 (10.3)
Range	30 to 76.6
Mean (SD) Hospital and Depression Scale scores	
Anxiety subscale	10.1 (4.9)
Depression subscale	7.5 (4.8)
Self-reported falls in previous year n (%)	
Fallen once	14 (22.2%)
Fallen more than once	28 (44.4%)
Physical activity level n (%) 5 missing	
Low	26 (44.8%)
Medium	21 (36.2%)
High	11 (19.0%)
Fear of Falling scale scores	
Modified Falls Efficacy Scale	
Mean (SD)	7.7 (2.7)
Median (IQR)	8.5 (5.7 to 8.5)
Mean (SD) Consequences of Falling Scale	31.3 (9.4)
Damage to Identity	17.8 (4.5)
Loss of Functional Independence	13.6 (5.5)
Modified Survey of Activities and Fear of Falling in the Elderly Scale	
Mean (SD)	25.9 (8.7)
Median (IQR)	23 (18 to 34)

Fear of falling scores: The mean score for the MFES [7.7 (SD 2.7); 95% CI 7.0 to 8.3] was lower than the threshold denoting ‘fear of falling’. For the MFES, a lower score denotes more fear of falling. In total, 30 (48%) of participants had a score lower than eight. Eleven of the 14 items had a mean score less than 8, of which the lowest was for confidence when using public transport. The mean COF score was 31.3 (SD 9.4; 95% CI 29.1 to 33.6), with the mean score for the Damage to Identity subscale being statistically significantly higher than the mean score for the Loss of Functional Independence subscale (17.8 (SD 4.41) vs 13.6 (SD 5.52); paired t-test 9.31, df62, *p* < 0.001). The highest individual item mean scores in the COF were for ‘I will be embarrassed’ (mean 3.4), ‘I will feel foolish’ (mean 3.2) and ‘I will be in pain’ (mean 3.1) in the Damage to Identity subscale. The mean score for the MSAFFE was 25.9 (SD 8.7; 95% CI 23.9 to 28.1) and the item with the highest mean score was avoid going ‘out when it is slippery’ (mean 2.2).

Comparison of fear of falling between fallers and non-fallers: Participants who reported a previous fall (*n* = 42) had significantly worse mean scores, suggesting greater fear of falling, on all three scales than those who had not previously fallen (*n* = 21) (Table 2). Both subscales of the COF showed worse scores in those who had fallen (Table 2) but the absolute and relative difference was greater for the Loss to Functional Independence subscale (15.2 vs 10.4; relative difference 50%) than for the Damage to Identify subscale (19.0 vs 15.3; relative difference 24%). Indeed, the biggest difference in the item scores was for the item ‘continue be active’, which is part of the Loss to Functional Independence subscale, but there was no significant difference between groups in the worst scoring items: ‘I will be embarrassed’ and ‘I will feel foolish’, which are part of the Damage to Identity subscale. A previous fall was not associated with age or body mass index in this sample (Table 2).

**Table 2:** Comparisons in demographic factors and fear of falling scores between self-reported fallers and non-fallers

Characteristic	Fallers *n* = 42 Mean (SD)	Non-fallers *n* = 21 Mean (SD)	Test of difference, *p* value 95% CI for difference
Mean (SD) BMI in kg/m^2^	43.8 (10.9)	39.8 (8.2)	t = 0.86, df61, *p* = 0.70
Age group in years			
18 to 35	13 (31%)	5 (24%)	Chi-square 1.29, df2,
35 to 44.9	12 (29%)	9 (43%)	*p* = 0.52
> =45	17 (40%)	7 (33%)	
Mean (SD) Modified Falls Efficacy Scale	7.0 (2.6)	9.0 (2.4)	t = 3.12, df61, *p* = 0.003 95% CI –3.4 to −0.7
Mean (SD) Consequences of Falling Scale	34.1 (8.6)	25.7 (8.6)	t = 3.67, df61, *p* = 0.001 95% CI 3.8 to 13.0
Mean (SD) Modified Survey of Activities and Fear of Falling in the Elderly Scale ^a^	28.8 (8.9)	20.1 (4.3)	t = 5.24, df60.9, *p* < 0.001 95% CI 5.4 to 12.3

^a^t test for unequal variances used, as appropriate; Median in fallers = 30 and in non-fallers = 18; Mann Whitney U test for difference in medians *p* < 0.001. *BMI* body mass index

Relationship between fear of falling and physical activity (Table 3): The relationship between fear of falling and physical activity was explored in the 58 women with activity data. MFES score was an independent predictor of low physical activity. There was a 35% increase in low activity with each unit decrease in MFES score after adjusting for age, BMI and depression (odds ratio = 0.65, 95% confidence interval 0.44 to 0.96, *p* = 0.03 – Table 3); none of the other factors were independent risk factors. After adjusting for the other factors, MSAFFE score was an independent predictor of low physical activity, with a 14% increase in low activity with each unit increase in MSAFFE score (odds ratio = 1.14, 95% confidence interval 1.01 to 1.28, *p* = 0.04). In addition, older age was also found to be an independent predictor, with a 5 times increase in odds for those aged 45 years or over compared to those aged less than 35 years (odds ratio = 5.1, 95% CI 1.04 to 24.55, p = 0.04) (Table 3). The overall COF score was not found to be an independent predictor of low physical activity (odds ratio = 1.08, 95% CI 0.99 to 1.18, *p* = 0.07). The MFES and MSAFFE remained independent predictors of low physical activity even when further adjusted for self-reported falls, the presumptive mediator of fear of falling [OR 0.57 (95% CI 0.36 to 0.91), OR 1.17 (95% CI 1.04 to 1.34)] (Table 3). Of the two subscales of the COF, a higher Loss to Functional Independence COF subscale score was significantly associated with low activity (odds ratio = 1.17, 95% CI 1.01 to 1.35, *p* = 0.033), when adjusted for all other factors.

**Table 3:** Logistic regression analysis of the relationship between each fear of falling measure and physical activity adjusting for all other factors (*N* = 58)

Measure	Characteristic	Odds ratio	95% CI
MFES	BMI per kg/m^2^	1.07	0.98 to 1.17
	Age group		
	< 35 years	1	
	35 to 44 years	1.62	0.46 to 10.6
	45 years and over	3.45	0.71 to 16.7
	Depression	0.93	0.77 to 1.14
	MFES score	0.65	0.44 to 0.96*
	As above + adjusting for self-reported fall		
	Fallen in last year	2.79	0.53 to 14.7
	MFES Score	0.57	0.36 to 0.91*
MSAFFE	BMI per kg/m^2^	1.06	0.98 to 1.16
	Age group		
	< 35 years	1	
	35 to 44 years	2.20	0.46 to 10.6
	45 years and over	5.06	1.04 to 24.5*
	Depression	0.90	0.72 to 1.12
	MSAFFE score	1.14	1.01 to 1.28*
	As above + adjusting for self-reported fall		
	Fallen in last year	2.53	0.54 to 12.0
	MSAFFE Score	1.17	1.03 to 1.34*
COF	BMI per kg/m^2^	1.06	0.98 to 1.15
	Age group		
	< 35 years	1	
	35 to 44 years	1.69	0.37 to 7.8
	45 years and over	5.23	1.11 to 24.5*
	Depression	0.96	0.80 to 1.16
	COF score	1.08	0.99 to 1.18
	As above + adjusting for self-reported fall		
	Fallen in last year	1.85	0.43 to 8.0
	COF Score	1.10	1.0 to 1.21

*BMI* Body mass index, *MFES* Modified Falls Efficacy Scale, *MSAFFE* Modified Survey of Activities and Fear of Falling in the Elderly Scale, *COF* Consequences of Falling Scale; *p < 0.05

## Discussion

In this study, a high proportion of obese women under 50 years of age reported having had a fall in the previous year. Others have reported a higher probability of tripping, slipping and falls among those who are obese compared to those who are not [26–29]. Talbot et al. reported a falls rate among women aged 20 to 45 of any body mass index that is a third of that seen in our study [30]. A population study of US adults reported that among women aged 45–79 the risk of a fall in the previous 12 months was higher among those who were obese [31]. It may not be surprising then, albeit a novel finding, that, in this study, obese younger women experienced high levels of fear of falling, irrespective of the measure used. For each measure of fear of falling, mean scores were similar to those observed in studies of elderly populations and other populations with balance issues [19, 20, 22, 32]. Similar to studies in the elderly, mean scores were significantly worse in obese younger women who had fallen in the last year compared to those who had not fallen [19–22].

Worse MFES and MSAFFE scores were associated with lower physical activity levels, potentially providing evidence for novel interventions to promote activity in this group. A recent Cochrane systematic review suggests that exercise interventions are probably effective in reducing fear of falling in community dwelling older adults [33], but there is less evidence available about the impact of psychological interventions. This was a cross-sectional study and the direction of effect is unclear. It could be that fear of falling leads to low physical activity because obese women avoid activities due to concerns about falling or it could be that low physical activity leads to more difficulties undertaking activities and, hence, concerns about falling if they undertake these activities. However, studies in the elderly have demonstrated that fear of falling precedes activity restrictions [8, 9]. While the concerns about Damage to Identity were higher than concerns about Loss of Functional Independence, fallers were more concerned than non-fallers about Loss of Functional Independence, and it was this subscale that was associated with low activity levels. These findings suggest that there may be a benefit to focussing on falls prevention and ongoing support after falls in obese younger adults to maintain activity levels.

Given that fear of falling encapsulates a number of different concepts [7], there are a number of available scales measuring a range of constructs but all developed for older people [18]. Considerable international effort has been focussed on developing a consistent measure, the Falls Efficacy Scale International (FES-I), which has excellent validity and reliability for elderly populations [34], and is increasingly used in fear of falling research. Some may criticise our study because we did not use this measure and relied on less rigorously validated tools. In our previous study comparing 8 obese young adults with 8 non-obese young adults, a small, albeit statistically significant, higher mean score on the FES-I was observed [35]. However, we felt that the activities included in this scale might not be of sufficiently high order to discriminate between groups, or cover all the concerns, of younger obese adults [36, 37]. The preceding qualitative study confirmed this and guided the choice of measures in this study. In addition, the activity avoidance measure was chosen specifically because, in part, the study was on the potential relationship between fear of falling and physical activity. Known group differences between fallers and non-fallers provides evidence of the validity of these tools in this population, but further work is needed to determine their reliability. The study was also limited to women. In part, this was because of the small number of men who accessed weight management services at that time, but also women have been shown to have higher levels of fear of falling in the elderly and women have higher rates of falls than men [31, 38]. However, future work should also look at these issues in men who are obese.

## Conclusion

Women who are obese and under 50 years of age report a high incidence of falls and fear of falling. Fear of falling appears to be associated with low levels of physical activity, although, the odds ratios were of borderline significance. This study was undertaken in a group who are already in contact with weight management services, but the findings suggest that further work is warranted to explore this important unique finding in larger, population-based cohort studies. Further elucidation will increase our understanding of the capacity of obese adults to undertake physical and incidental activities and suggest novel interventions to promote lifestyle changes and weight management or through falls prevention to reduce activity restriction. This may require the development and validation of more relevant measures for this population.

## Abbreviations

BMI: Body mass index
COF: Consequences of Falling Scale
FES-I: Falls Efficacy Scale International
MFES: Modified Falls Efficacy Scale
MSAFFE: Modified Survey of Activities and Fear of Falling in the Elderly Scale
SF-IPAQ: Short Form International Physical Activity Questionnaire
